# A stacking ensemble classifier-based machine learning model for classifying pollution sources on photovoltaic panels

**DOI:** 10.1038/s41598-023-35476-y

**Published:** 2023-06-24

**Authors:** Prince Waqas Khan, Yung Cheol Byun, Ok-Ran Jeong

**Affiliations:** 1 School of Computing, Gachon University, 1342 Seongnam-daero, Seongnam, 13120 Republic of Korea; 2 Department of Computer Engineering, Jeju National University, Jeju, 63243 Republic of Korea; 3 Department of Computer Engineering, Major of Electronic Engineering, Jeju National University, Institute of Information Science and Technology, Jeju, 63243 South Korea

**Keywords:** Energy science and technology, Engineering

## Abstract

Solar energy is a very efficient alternative for generating clean electric energy. However, pollution on the surface of solar panels reduces solar radiation, increases surface transmittance, and raises the surface temperature. All these factors cause photovoltaic (PV) panels to be less efficient. To address this problem, a stacking ensemble classifier-based machine learning model is proposed. In this study, different sources of pollution on each solar panel are used, and their power generation is recorded. The proposed model includes gradient boost, extra tree, and random forest classifiers, with the extra tree classifier serving as a meta-learner. The model takes into account various weather features during the training process, including irradiance and temperature, aiming to increase its accuracy and robustness in identifying pollution sources on the PV panel. Moreover, the proposed model is evaluated using various methods in order to examine performance metrics such as accuracy, F1 score, and precision. Results show that the model can achieve an accuracy score of 97.37%. The model’s performance is also compared to state-of-the-art machine learning models, demonstrating its superiority in accurately classifying pollution sources on PV panels. By utilizing different sources of pollution and weather features during training, the model can accurately classify different pollution sources, resulting in increased power generation efficiency and the longevity of PV panels. The main results of this study can be used to manage and maintain PV panels since the model can identify PV modules that need to be cleaned to keep producing the most power. Furthermore, the efficiency, reliability, and sustainability of PV panels can be further enhanced by the proposed model.

## Introduction

Renewable energy sources have the potential for effective advancement and sustainable growth. The importance of using renewable energy sources holds a unique position in the realm of contemporary technological development^1^. The utilization of solar power represents a highly promising opportunity for the production of energy in the near future, primarily due to its capacity to serve as a renewable natural energy source, which ranks among the largest of such sources in the world and can be harnessed for distribution beyond the demands of local consumption^2,3^. It does not require additional costs; it is clean and does not pose a risk of contamination. Sustainable development requires methods and tools to measure and compare the environmental impact of human activities on different products, services, etc. Photovoltaic (PV) systems tend to be a promising energy source to save energy and reduce CO2 emissions. To further increase solar energy efficiency, the size of the materials used in the solar system and the system itself are designed to maximize energy requirements and for recycled materials that reduce greenhouse gas (GHG) emissions^4^.

Solar energy is a good way to make electricity as it mostly comes from a clean source; however, adjustable and unadjustable aspects can affect a PV module’s efficiency. One such aspect is pollution on the surface of the solar panel, which reduces solar radiation, attenuates the incoming solar irradiance, and reduces the efficiency of PV panels by increasing their temperature. According to Sweerts et al.^5^ due to pollution, PV potential decreased on average by 11–15% between 1960 and 2015. The power output of an optical voltage module is dependent on the amount of radiation reaching the solar cell. Hence, various factors can influence the ideal or optimal output of an optical voltage module^6^. However, the environment is a critical parameter that directly impacts the performance of the optical voltage module. Specifically, the accumulation of pollution on the surface of the PV panel can affect the total power supplied by the PV module on a daily, monthly, seasonal, and yearly basis. Solar panels are composed of two interconnected parameters that can be affected by waste collection properties, pollution properties, and the local environment. These pollution stains, such as leaves and dirt, can block some cells in the PV module, causing a decrease in efficiency and a reduction in lifespan, negatively impacting overall system performance, leading to permanent module staining, and potentially voiding the warranty^7^. Regular cleaning is essential to ensure maximum output, maintain the warranty, save money, and prevent permanent module staining^8^. Dust is one of the most critical environmental factors affecting the performance of solar panels and belongs to the category of irreversible factors. It can reduce the performance of PV panels by causing physical damage, reducing incoming solar radiation, increasing the temperature, and altering the electrical properties of the panel^9,10^. The degree of depreciation depends mainly on the deposit concentration and is controlled by several factors. Dust reduces solar radiation, reduces surface transmittance, produces a mild shading effect, and reduces the performance of PV panels by increasing panel temperature. The degradation performance is linear with the concentration of dust deposits^11^.

The accumulation and deposition of aerosol particles in PV panels, commonly referred to as “PV panel soiling, ”affects the performance of the PV power system. To demonstrate the efficiency of PV systems and create cost-effective mitigation, soil impact assessments were recommended at different locations and times. Machine learning (ML) is widely used for a variety of purposes in the renewable energy industry^12–14^. Some of the applications involve energy forecasting^15^, solar radiation forecasting^16^, locations and sizes of solar^17^, and roof shape classification^18^. We have proposed to use ML for pollution source classification.

This study employed an ML model based on stacking ensemble classifiers, including gradient boost, extra trees, and random forest classifiers, to identify the source of PV panel pollution. The extra-tree classifier was used as the meta-learner of the stacking ensemble model. Various weather features were considered during the model training process. The performance of the proposed model was evaluated using metrics such as accuracy, precision, and F1 score and compared to state-of-the-art machine learning models. The solar panels were installed according to the six modules set aside for the experiment. Different sources of pollution have been used on each solar panel, and their power generation has been recorded. The major contributions of this article can be summarized as follows:

- Six modules were dedicated to the installation of solar panels for experimental purposes, where each panel was subjected to different sources of pollution.
- A machine-learning model based on stacking ensemble classifiers, including gradient boost, extra trees, and random forest classifiers, was employed to classify the source of pollution on the surface of the PV panel.
- The training of the model included consideration of weather features and its performance was evaluated using various metrics.
- The proposed approach has demonstrated superior performance compared to state-of-the-art machine learning models in terms of accuracy and enhancing the sustainability of PV panels.

The remainder of the article is organized into five main sections, starting with the “Literature review” section. The “Proposed approach” section describes the machine learning model that is proposed for classifying pollution sources on photovoltaic (PV) panels. The next section, “Case study,” presents a case study of the proposed approach and analyzes the collected data. This section includes a detailed explanation of the data collection process and the methods used to analyze the data. The “Results” section presents the case study’s experimental results, including the proposed model’s performance using evaluation functions such as accuracy, F1-score, and precision. Finally, in the “Conclusions” section, the study’s main findings are summarized, and the implications of the proposed model for the management and maintenance of PV panels are discussed. Potential future research directions to improve the proposed model and its application to real-world scenarios are also suggested.

## Literature review

Pollutants in the air, like dust, smog, and small particles, can settle on solar panels and form a layer of grime that keeps sunlight from reaching the photovoltaic cells. This causes the solar panel’s energy output to go down, which can significantly affect how much energy a solar power system makes as a whole. Researchers have used machine learning and deep learning techniques to monitor soiling losses in photovoltaic systems.

The paper by Heinrich^19^ addresses the issue of soiling losses in photovoltaic systems, which is a significant concern for remote power systems. The authors propose a low-cost monitoring system for cleaning interventions on photovoltaic modules during the daytime, which can be helpful in deciding whether additional cleaning operations are necessary. The problem is formulated as a classification task using a time window of a photovoltaic array’s temperature, voltage, and current measurements. The authors investigate machine learning tools based on logistic regression, support vector machines, artificial neural networks, and random forests to achieve the classification task. In the study by Martin et al.^20^, the authors focused on two methods for identifying soiling in residential solar installations. The first method calculates a daily energy loss due to soiling by comparing two calculated power curves: the expected best-case scenario curve and a weather-corrected curve, which estimates the day’s power curve in the absence of cloud cover. The second method uses a multilevel k-means clustering strategy to compare sites’ performance in the same weather region. The study found that these methods do not require feature-rich datasets but can operate solely on time-series power values. The second method yielded promising results, verified by ground truth observations. By plotting these classifications over time as a heatmap, the researchers could identify the worst offenders and verify that these sites were dirty. The study concludes that with further work, the proposed system could be developed into an autonomous system for alerting homeowners when their solar panels require cleaning.

Deep learning techniques like convolutional neural networks (CNNs) can be used to recognize and process images taken by cameras in solar farms to find and measure soiling and other environmental factors that affect solar panels. The work by Liu et al.^21^ discusses the importance of identifying the various types of soiling on PV panels for solar energy systems, as it can cause significant energy loss. The study presents a CNN model with nine layers to classify panel images into six categories of soiling. The dataset used for training and testing the model is split into categories, and the experimental results show good performance, with an average accuracy of 0.98%. Their model can aid in determining countermeasures to prevent power waste by identifying the features of soiling. They also propose extending the model to solve power loss prediction using object detection and semantic segmentation. The paper concludes that cleaning is essential to maintaining solar power systems, and the CNN model is a powerful and simple method for image classification. Overall, the study highlights the significance of identifying and classifying different types of soiling on PV panels and the potential of using machine learning methods like CNN to help maintain solar energy systems. In work by Zhang et al.^22^, the authors proposed a probabilistic quantification method called SolarQRNN to estimate the power loss caused by soiling on solar PV panels. The method utilizes images captured by surveillance cameras and combines quantile regression and computer vision models. The proposed method employs a novel quantile loss function and deep learning structures based on residual convolution units. The method was tested on a solar panel soiling image dataset, and the results showed that SolarQRNN outperformed benchmark classification models with at least 51% improvements in evaluating metrics. The proposed method provides a probabilistic estimate of power loss caused by soiling, which can help improve the accuracy of solar PV power generation forecasting and scheduling. The paper’s authors, Yang et al.^23^, propose a method to quantify soiling using images on large solar photovoltaic (PV) panel arrays. Soiling caused by dust accumulation is a significant challenge facing large-scale solar PV plant operations, especially in arid regions. Traditional methods of assessing and mitigating soiling effects are limited by area coverage and the frequency of data collection. To overcome this limitation, the authors developed a prototype lab system and image processing algorithm to use images to quantify soiling on PV panels directly. Lab tests are conducted to determine the impacts of various camera settings and viewing angles by calculating the black-and-white ratio from photos of a surrogate surface with controlled dust loadings. The experiments reveal that dust loading can be quantified under optimal imaging conditions, suggesting that aerial photos may one day be used to quantify soiling on PV panels. Convolutional neural networks (CNN) are presented as a unique method for analyzing solar panel soiling and defects in the paper by Mehta et al.^24^. The suggested method makes predictions about power loss, soiling localization, and soil type using an RGB image of a solar panel and environmental parameters. There are four steps to the process, and all you need to get started is a set of panel photos with power loss labels. Web-based supervised learning is used to categorize the projected localization masks’ impact area into distinct soiling categories. In order to enhance CNNs’ capacity for localization, this work presents a unique bi-directional input-aware fusion (BiDIAF) block. The method is scalable and performed admirably on solar panel photos scraped from the internet.

In the manuscript by Chuluunsaikhan et al.^25^, the authors proposed a model to predict the power output of solar panels based on weather and air pollution features. Five sets of features were experimented with six machine-learning methods. The authors demonstrated that solar panel features achieved the best accuracy of around 95% in all models. Weather and top-correlated features showed similar good results. However, air pollution features did not perform as well compared to the other experiments. The best model was created in the form of an RF model with around 98% accuracy. In this study by Jia et al.^26^, the authors assessed the performance of three commonly used machine learning models for predicting global and diffuse solar radiation in eight Chinese cities representing different geo-climatic and pollutant conditions. The support vector machine (SVM) outperformed the other models in all locations, followed by generalized linear modeling (GLMNET) and random forest (RF). The accuracy of solar radiation prediction was closely related to the weather and pollution conditions, and the SVM model demonstrated its reliability in radiation prediction under slight pollution and stable weather conditions.

The proposed approach addresses research gaps by providing experimental data on pollution impact on solar panels, using ensemble learning for accurate classification of pollution sources, considering feature selection to enhance model accuracy, and improving PV panel sustainability through identification and mitigation of pollution sources.

## Proposed approach

**Figure 1:**
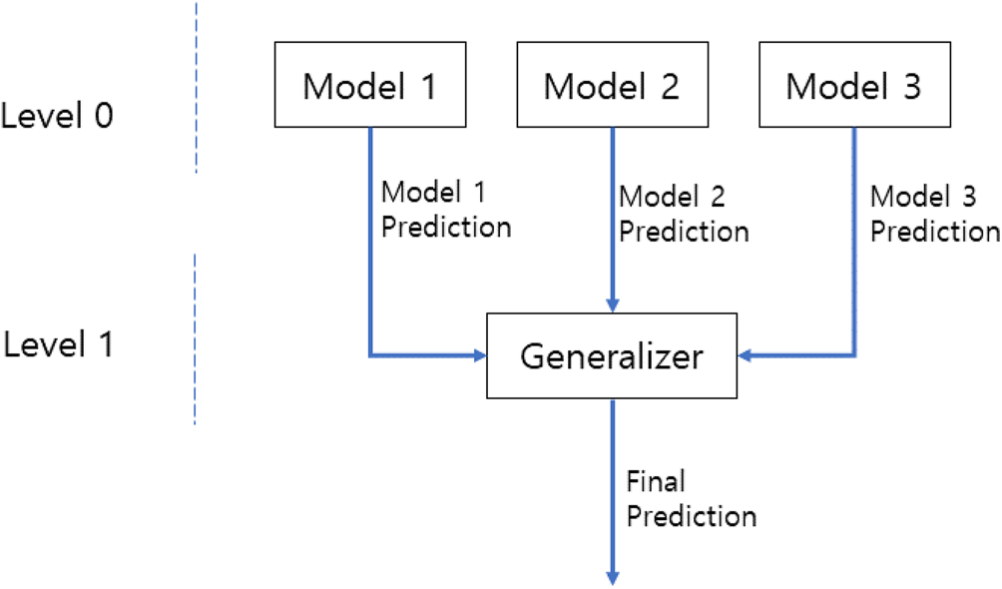
Stacking ensemble model.

Stacking is a type of ensemble learning that uses multiple classification or regression models to make predictions that are more accurate. In this technique, the output of several base models is used as input to a meta-model, which makes the final prediction. The base models are trained on the input data and generate their individual predictions. These predictions are then combined and used as inputs to the meta-model, which learns from them to make a final prediction^27^. Stacking allows combining the strengths of several models and producing a more accurate and robust prediction than any single model alone. Figure 1 provides a general diagram of the stacking ensemble model. The proposed stacking ensemble involves using a set of three base models to generate outputs at Level 0. These three outputs are used as inputs in the level 1 classifier. A level 1 classifier is called a meta-model. Level 0 classifier is called the base model^28^.

**Figure 2:**
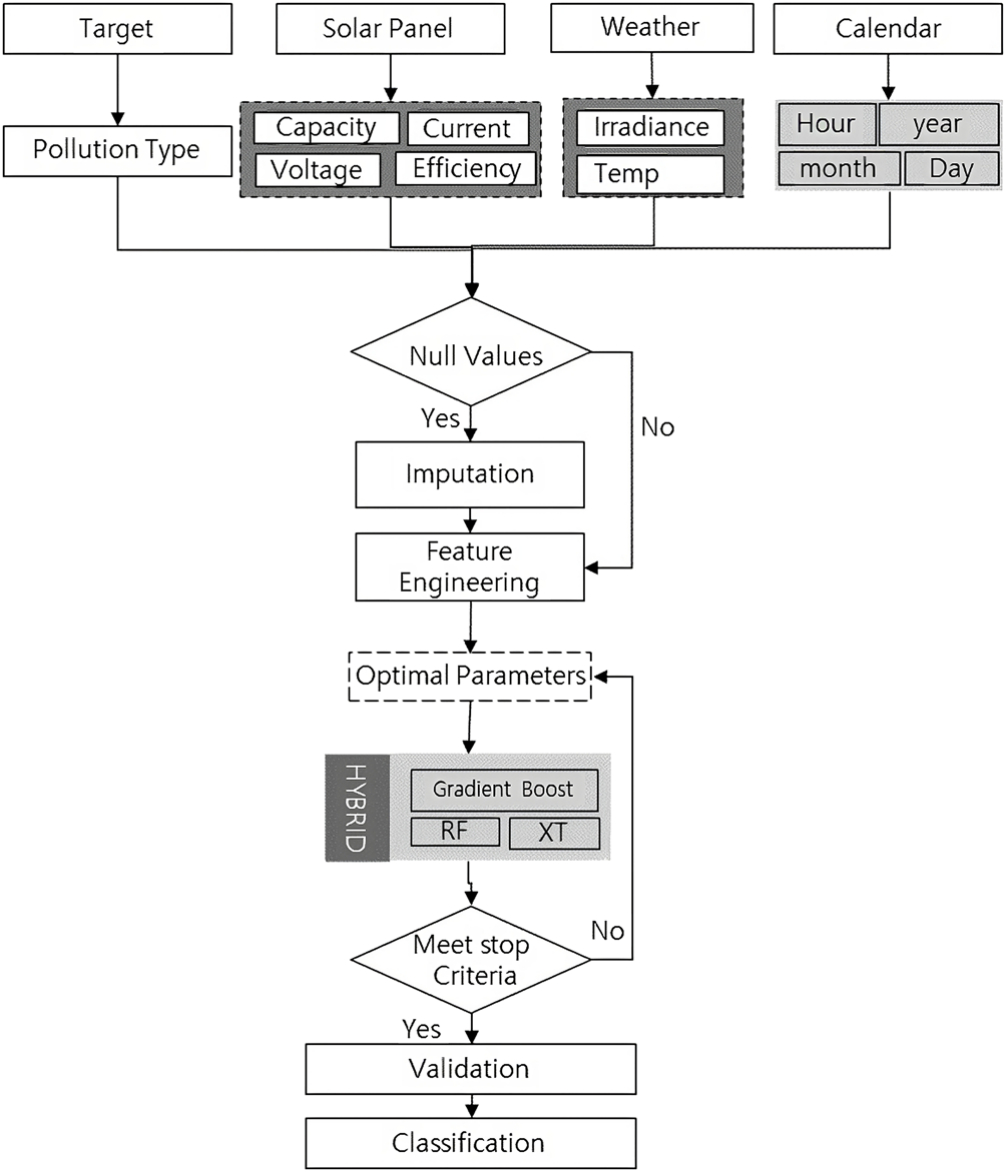
Flow chart of proposed methodology.

The proposed methodology involves configuring a stacking ensemble model that utilizes three machine learning models: gradient boost, extra tree, and random forest. The first level of the model consists of Gradient Boost, Extra Tree, and Random Forest, while the second level consists of the Extra Tree model. The flowchart of the methodology is shown in Fig. 2. The input data consists of target values for solar panel features and calendar features. The data collection process involves the extraction of input features from the solar panels, including capacity, voltage, and current. Additionally, weather factors, such as irradiance and temperature, are measured and recorded. Time features such as hour, year, month, and day are also included as input features to capture temporal variations. The first step involves checking for null values; if there are any, they are imputed. Otherwise, feature engineering is performed. The hybrid algorithm that generates the input features consists of gradient boost, random forest, and extra tree algorithms. The output of these algorithms is combined using a stacking ensemble classifier. Validation is performed using different evaluation metrics. Finally, the classification prediction is made, as shown in the last step of the flowchart.

Gradient boosting is a machine learning method for regression and classification problems that builds a predictive model, usually in the form of a decision tree, with a weak set of predictive models. Strengthening the extreme layout creates a forest of trees, but it does it in an extra way. The algorithm regenerates trees, minimizes errors, and reduces the best predictions to tree sets^29^. Learned trees are retained, and new trees are added to reduce the target function or prediction error. The trees are grown sequentially; each tree is grown using information from previously grown trees. Each tree corresponds to the latest version of the original data set based on a prefabricated tree.

**Figure 3:**
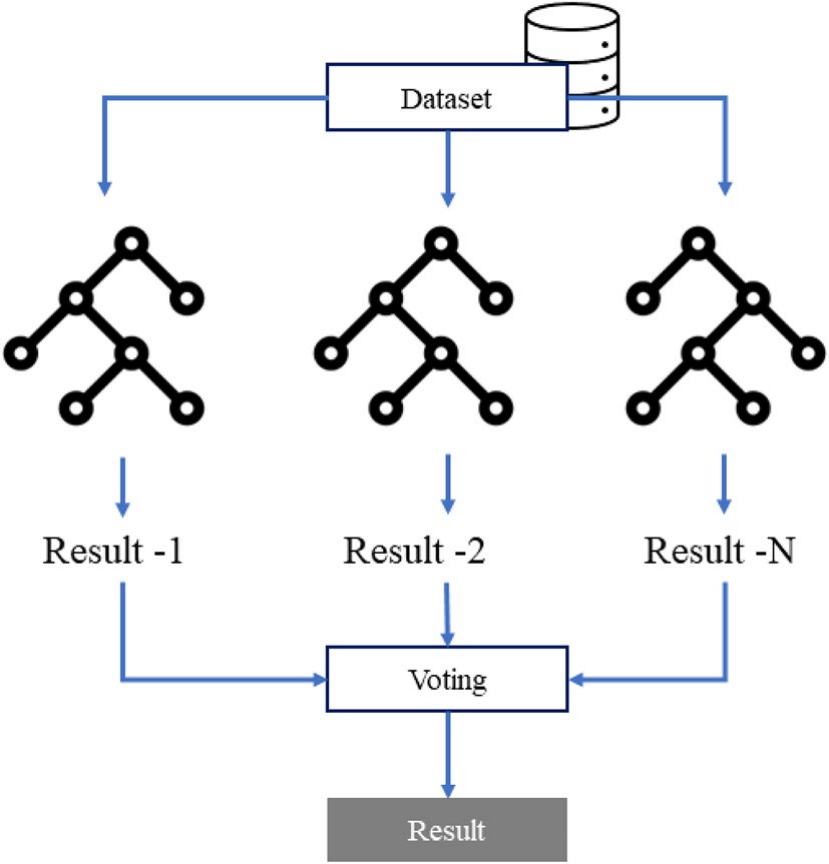
Random forest model.

Random forest is a widely used ensemble learning method in which a classification conclusion is obtained by aggregating the results of multiple decision trees generated during training^30^. The general architecture of a random forest model is illustrated in Fig. 3. Extra trees are similar to random forests in that they train multiple decision trees, but they differ in that they randomly select features among all available features to partition nodes. In contrast to random forests, extra trees do not use bootstrap samples or duplicate training samples; they use the entire training set to construct each decision tree. Furthermore, rather than finding the best split when splitting, extra trees inject randomness by setting the number and characteristics of data samples^31^.

**Figure 4:**
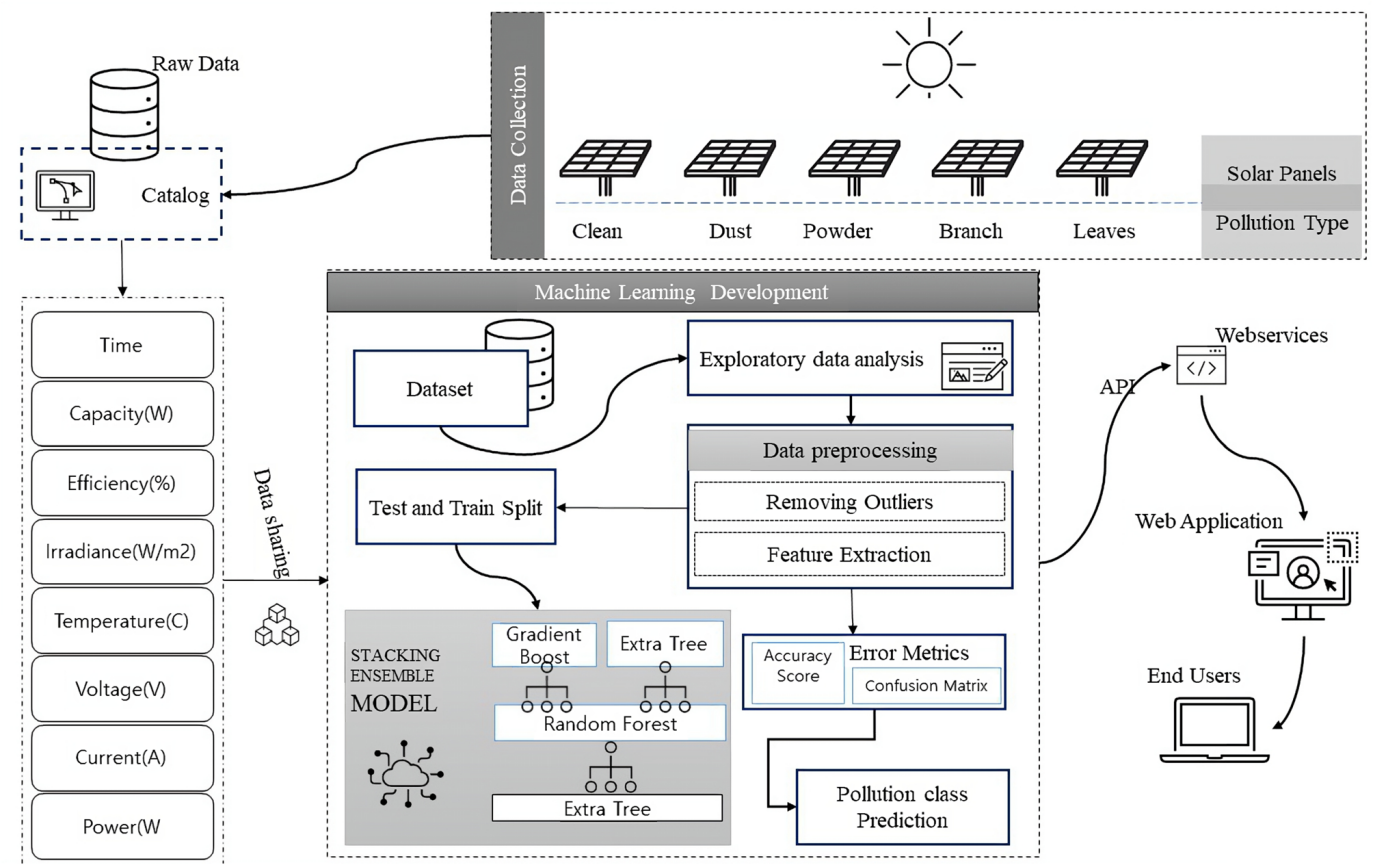
Overall structure of the proposed system.

Figure 4 represents the general structure of the suggested system. Five different solar panels were used to collect data. Each solar panel was manually polluted using different pollution types. That includes leaves, dust, powder, and branches. The solar power company collects this data. This data contains different types of information, such as module ID, experiment ID, irradiance, temperature, voltage, current, and power. Each solar panel’s capacity and efficiency are also provided with the dataset. The data were analyzed using different exploratory data analysis techniques. Raw data is preprocessed using different techniques, including removing outliers and null values and using feature extraction methods. Solar panels are installed according to the six modules set in the original data set. And then, different experiments are performed with different sorts of pollution. The module ID column consists of the module number, and the Exp ID column consists of the experiment performed. These two columns have been removed because no module ID or EXP ID will be provided as input when implementing the proposed system. Then preprocessed data is divided into training and test sets. Training data is given to the ML model, which comprises a stacking ensemble learning model. This model contains three algorithms: a gradient boosting, an extra tree classifier, and a random forest classifier. The presented model is evaluated using various evaluation metrics, including an accuracy score and a confusion metric. The output of this model can be accessed by end-users using a graphical user interface (GUI) that gets the information from the deployed module using an application programming interface (API) on a web service over the hypertext transfer protocol secure (HTTPS) protocol.

**Table 1:** Units of variables.

Sr#	Variable	Unit
1	Capacity	W
2	Efficiency	%
3	Irradiance	W/m2
4	Temperature	C
5	Voltage	V
6	Current	A
7	Power	W

**Figure 5:**
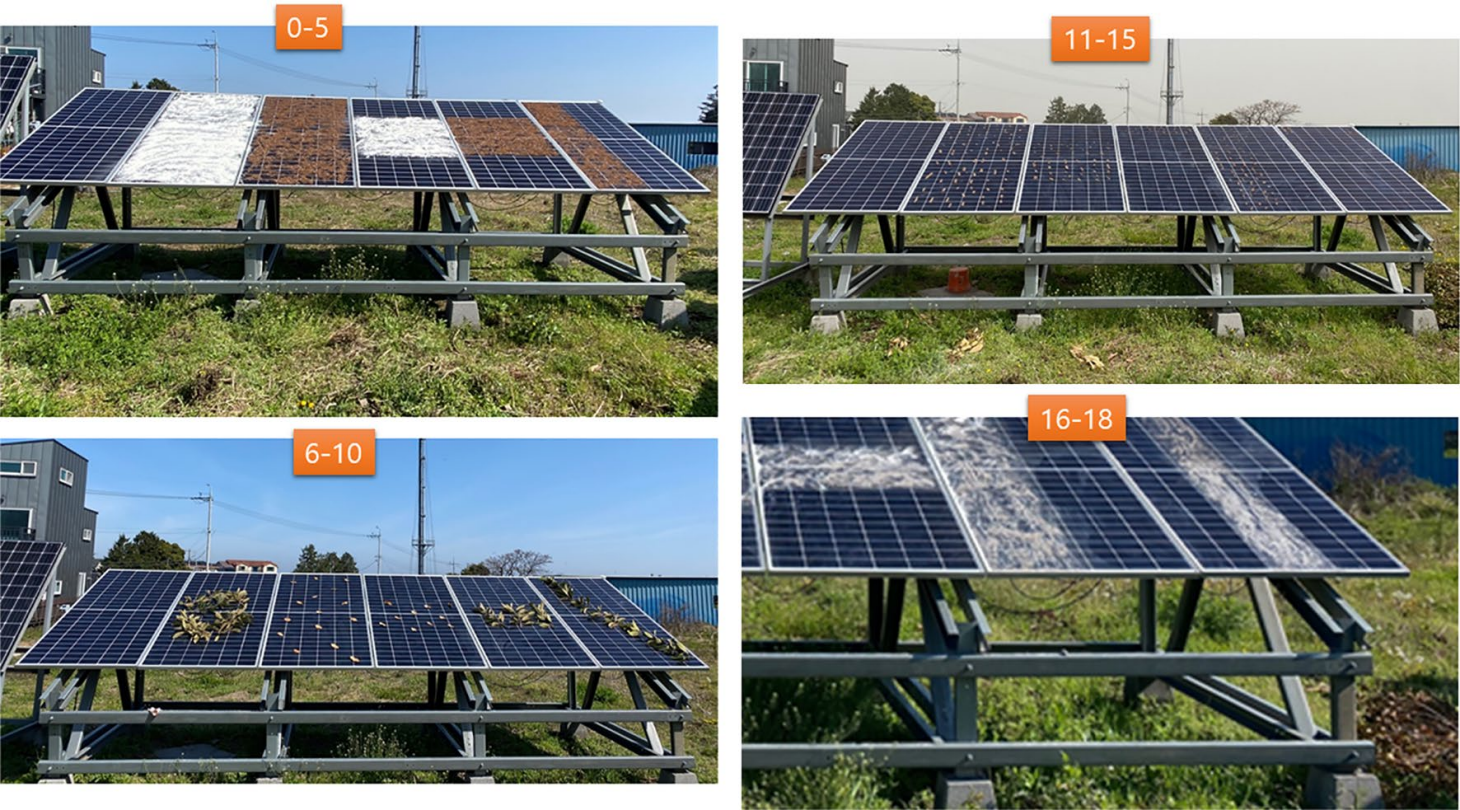
Solar panel testbed setup for experimental purpose.

## Case study

Solar panels were set up according to the six modules set up to get the data, and then different pollution types were used in different experiments. Figure 5 shows the installation location picture of the solar panel setup. The dataset has 12 variables named as $Module_ID$, Capacity, Efficiency, Irradiance, Temperature, $Exp_ID$, Voltage, Current, Power, Module, and Type. The total number of records (rows) is 72143, with no missing or duplicate values. Capacity (W) has a constant value of “420”, and Efficiency(%) has a constant value of “19.6”. Irradiance (W/m2) has 3154 (4.4%) zeros, and temperature (C) has 1155 (1.6%) zeros. Table 1 provides detailed information about the dataset variables, including their units. There are six numeric variables, namely capacity, efficiency, irradiance, temperature, voltage, and current, and their units. One variable, namely date-time, is of the data type date-time. Additionally, the dataset has five categorical variables. The table serves as a reference guide for understanding the dataset’s units and types of variables.

**Figure 6:**
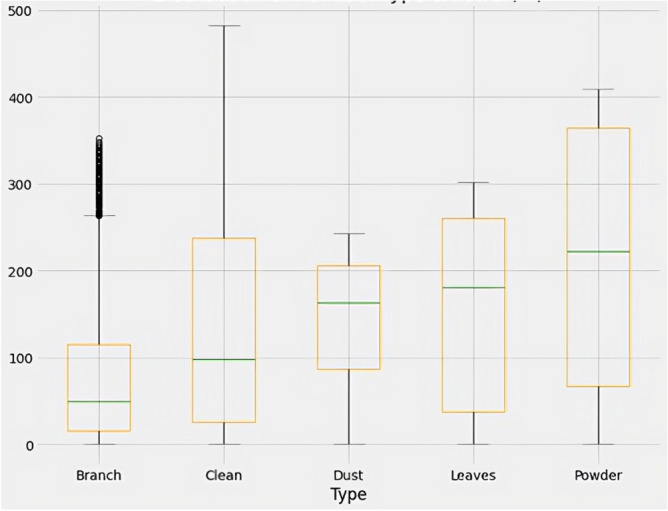
Box-plot of power according to pollution type.

**Figure 7:**
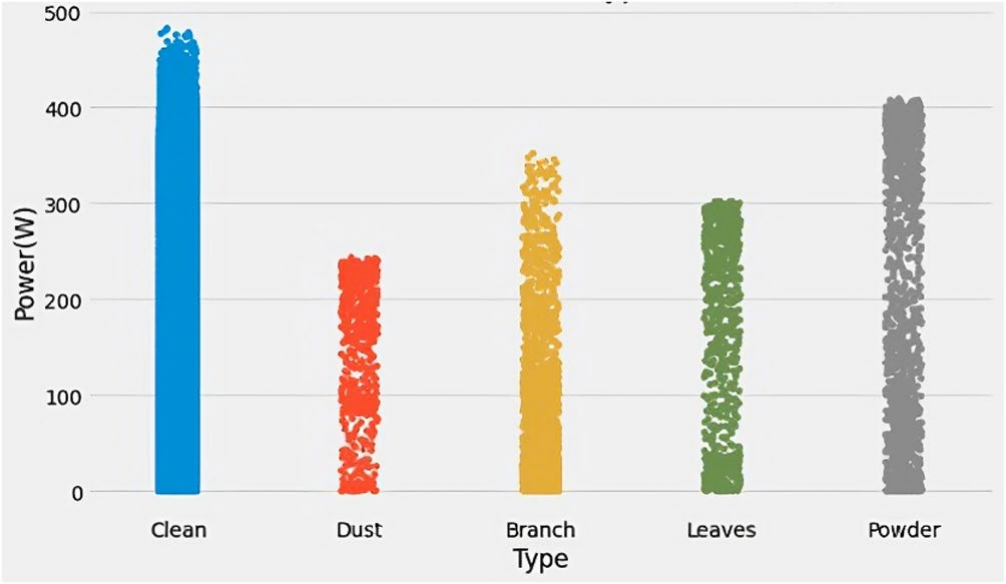
Power distribution according to pollution type.

Figure 6 displays the box plot of power with respect to pollution type. The branch pollution category exhibits the highest number of outliers, suggesting that the power generation from panels exposed to branch pollution is more variable and less consistent than other pollution types. Additionally, Fig. 7 depicts the distribution of power generated by the solar panels as per the type of pollution. It is observed that dust pollution causes the least power generation, whereas clean panels produce the highest power. This finding aligns with previous studies that have reported reduced efficiency and output due to the accumulation of dust on solar panels. Thus, the box plot and power distribution graphs provide valuable insights into the relationship between pollution type and solar panel performance.

**Figure 8:**
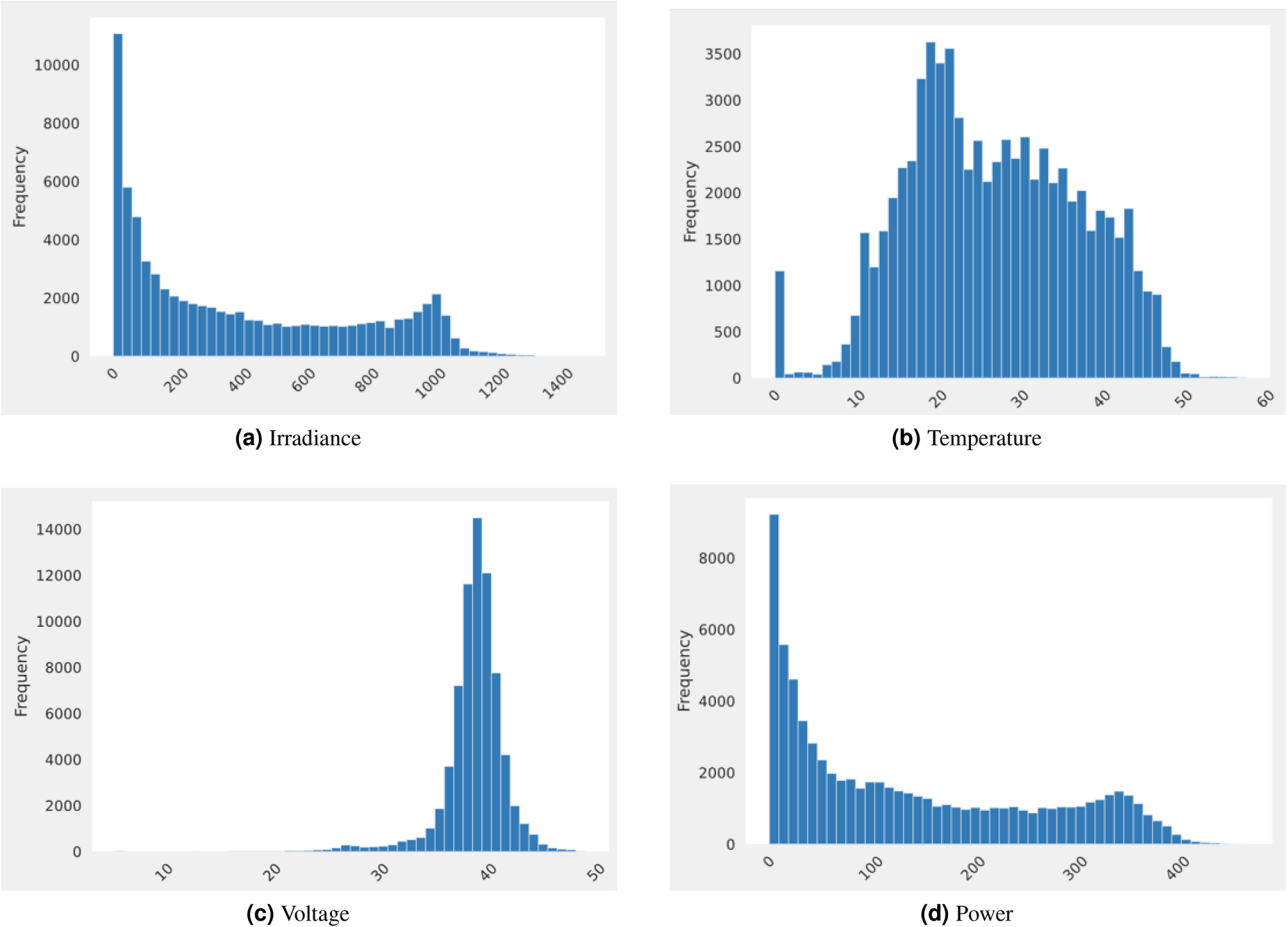
Count for each parameter.

Figure 8 shows the count for different weather and solar panel parameters. Figure 8a represents the frequency of irradiance values. Most irradiance values lie between 0 and 200, while the least lie between 1000 and 1473. It has a minimum value of 0 and a maximum value of 1473. Figure 8b frequency count of temperature values. It has a minimum value of 0 and a maximum value of 57.6. Most of the temperature values lie between 10 and 30, while the least is between 50 and 57.6. Figure 8c frequency count of voltage values It has a minimum value of 0 and a maximum value of 1473. Most of the voltage values lie between 35 and 45, whereas the least values lie between 0 and 20. It has a minimum value of 5.42 and a maximum value of 48.94. The frequency count of power values is provided by Fig. 8d. It has a minimum value of 0.003 and a maximum value of 468.65. Most of the power values lie between 0 and 100, whereas the least is between 400 and 469.

**Figure 9:**
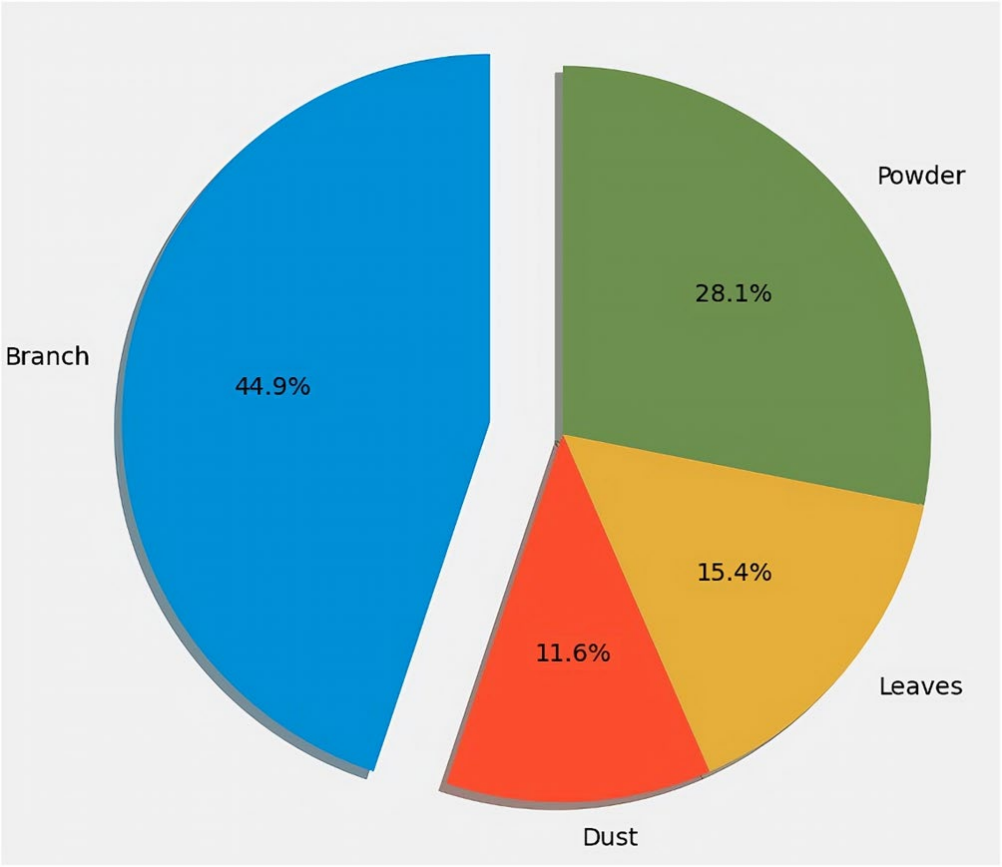
Pollution type distribution other than clean panel data.

Figure 9 depicts the pollution type distribution other than clean panel data. Where it is observed that a branch-polluted solar panel has 44.9% of data. In contrast, the powder-polluted solar panel has 28% data, the leaf-polluted solar panel has 15% data, and the dust-polluted solar panel has 11.6% data.

The original data set includes six modules for installing solar panels, followed by various experiments with different types of pollution. The module ID column denotes the module number, while the Exp ID column indicates the experiment performed. However, since the proposed system will not require module ID or EXP ID as input during implementation, these two columns have been eliminated from consideration.

## Experimnetal results

**Figure 10:**
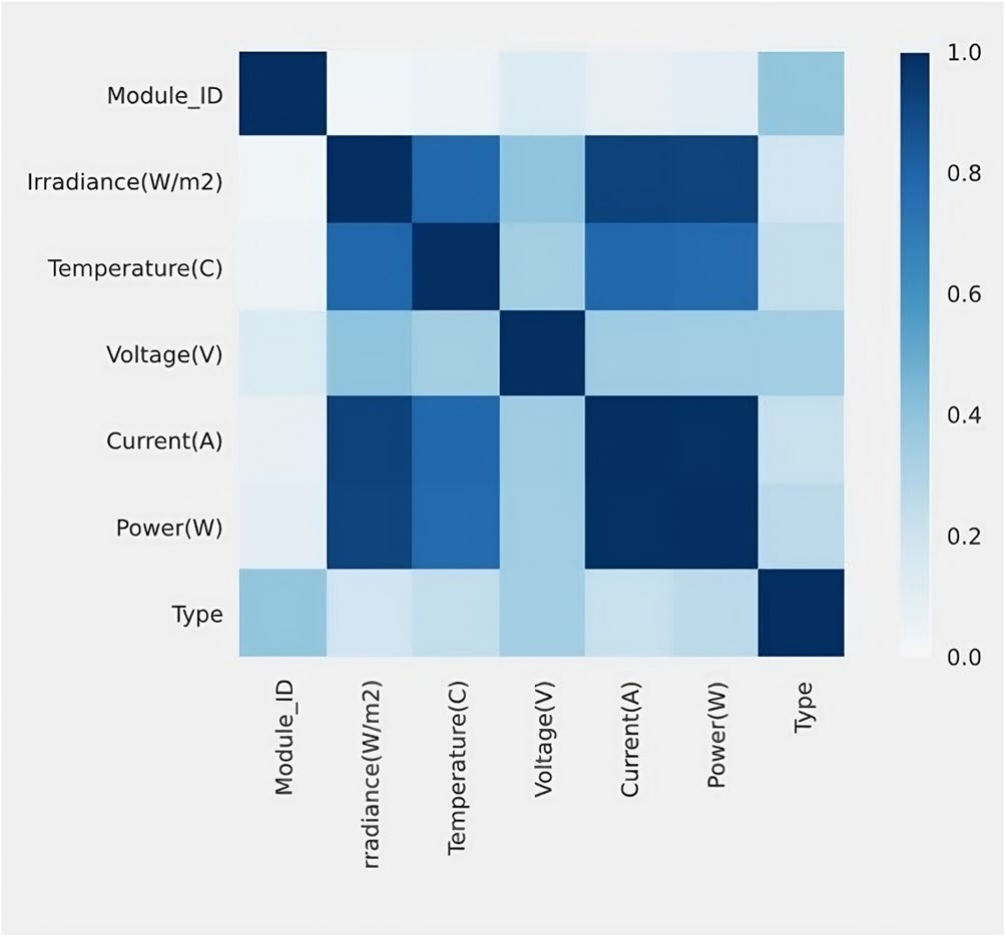
Correlation diagram.

This section covers the experimental results achieved using the presented approach. The proposed model has been compared with state-of-the-art algorithms. Phik Correlation is a new and practical correlation coefficient^32^. It works consistently between categorical, ordinal, and interval variables. It captures non-linear dependency and reverts to the Pearson correlation coefficient in the case of a bivariate normal input distribution^33^. Figure 10 shows the correlation diagram of features. It is observed that irradiance (W/m2) is highly correlated with temperature (C). Temperature (C) is highly correlated with irradiance (W/m2). The current (A) is highly correlated with irradiance (W/m2). Power (W) is highly correlated with irradiance (W/m2). Type is highly correlated with efficiency (%), and module ID is highly correlated with efficiency (%).

**Figure 11:**
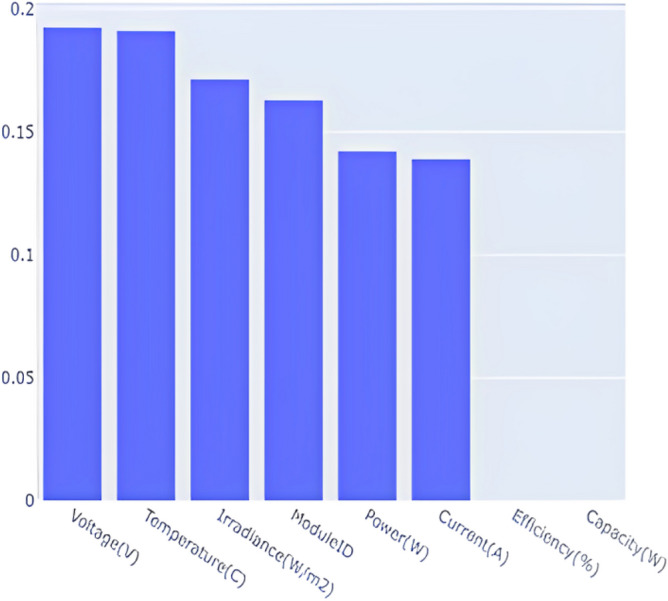
Feature importance.

Feature importance is a machine learning method that is often used to figure out which features are best for making predictions. In this context, it helps us understand which solar panel features have the greatest impact on the model’s ability to classify pollution sources. Figure 11 displays the feature importance diagram for the trained model. Efficiency and capacity features are shown to have zero importance, which indicates that they do not significantly contribute to the performance of the model. As a result, these columns were removed from the dataset, and the model was retrained. This new figure shows the feature’s importance after the removal of the efficiency and capacity columns. This approach allows us to focus on the most important features that have a greater impact on the model’s accuracy.

**Figure 12:**
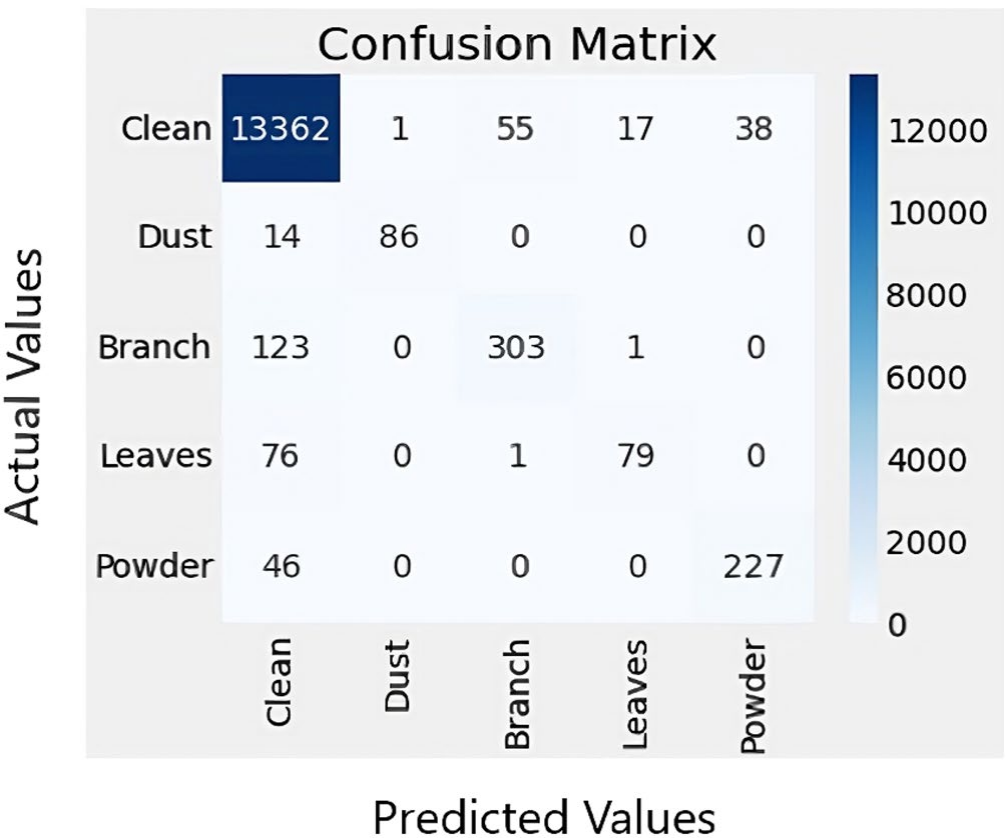
Confusion matrix.

The confusion matrix is a vital tool to evaluate the performance of a classification algorithm^34^. It summarizes the number of correct and incorrect predictions made by the model. The matrix contains four essential values: true positive (TP), true negative (TN), false positive (FP), and false negative (FN). These values help in calculating different performance metrics, such as accuracy, precision, recall, and F1-score. In this study, the confusion matrix is used to evaluate the performance of the proposed model. Figure 12 represents the confusion matrix obtained after applying the model. Each column of the matrix corresponds to a predicted class, while each row relates to an actual class. A perfect model that correctly classified all the complaints would result in the confusion matrix having values only in the diagonal. However, in practical scenarios, there are bound to be some errors, resulting in off-diagonal values. The confusion matrix is an essential tool to identify the type and frequency of errors and improve the model’s performance accordingly.

In the context of multi-class and multi-label classification problems, precision, recall, and F1 score are crucial metrics for evaluating the performance of classification models^35^. The precision, recall, and F1 score for each class is computed, as shown in Table 2. The precision measures the proportion of correctly classified instances among the predicted instances, while the recall measures the proportion of correctly classified instances among the actual instances. The F1 score is a harmonic mean of precision and recall, providing a balanced measure between the two metrics. Equations 1, 2, and 3 were used to compute the precision, recall, and F1 score, respectively. The precision, recall, and F1 score were calculated for each class: clean, dust, branches, leaves, and powder. The results in Table 2 indicate that the model performed exceptionally well for the Clean, Dust, and Powder classes, achieving F1 scores of 0.99, 0.92, and 0.84, respectively. However, the model’s performance was less accurate for the Branch and Leaves classes, achieving F1 scores of 0.78 and 0.63, respectively.

1 $$\begin{aligned} Precision= & {} \displaystyle \frac{TP}{TP+FN} \end{aligned}$$

2 $$\begin{aligned} Recall\ {}= & {} \frac{TN}{TN+FP} \end{aligned}$$

3 $$\begin{aligned} F1 Score\ {}= & {} \frac{2*Precision*Recall}{Precision+Recall} \end{aligned}$$

**Table 2:** Precision, Recall and f1-score of each class.

Sr #	Class	Precision	Recall	f1-score
1	Clean	0.98	0.99	0.99
2	Dust	0.99	0.86	0.92
3	Branch	0.85	0.72	0.78
4	Leaves	0.82	0.51	0.63
5	Powder	0.86	0.83	0.84

The accuracy of a classification model is a crucial factor in evaluating its performance. This study compares the proposed stacking ensemble classifier model’s accuracy with state-of-the-art classification algorithms such as extra trees, random forests, gradient boosting, K neighbors, catboost, ADA boost, bagging KNN, decision trees, and voting classifiers. We calculated the accuracy using Eq. 4, where TP, TN, FP, and FN represent True Positive, True Negative, False Positive, and False Negative, respectively^36^.

4 $$\begin{aligned} Accuracy = \frac{TP+TN}{TP+TN+FP+FN} \end{aligned}$$

Table 3 shows the comparison with state-of-the-art algorithms. The proposed stacking ensemble classifier model achieved the highest accuracy of 97.37%, outperforming all the other algorithms. Extra trees and the voting classifier performed well, achieving 96.36% and 96.17% accuracy, respectively. On the other hand, bagging KNN had the lowest accuracy of 80.31%. These results demonstrate the effectiveness of the proposed approach in accurately classifying the pollutant type.

**Table 3:** Accuracy comparison with state-of-the-art algorithms.

	Model	Accuracy (%)
1	Extra trees	96.36
2	Random forest	95.13
3	Gradient boosting	94.29
4	K neighbors	92.04
5	Cat boost	93.45
6	Ada Boost	91.31
7	Bagging KNN	80.31
8	Decision tree	86.28
9	Voting classifier	96.17
10	Proposed	97.37

## Discussion and conclusions

Solar, wind, and hydropower are renewable energy sources that can generate clean electricity. Numerous factors limit a solar panel module’s perfect outcome or optimum output. The environment is one of the contributing factors that directly affects photovoltaic performance. Solar panel surface pollution reduces solar radiation and raises panel temperature, thus reducing PV panel performance. Appropriate measures may be proposed earlier to eradicate or decrease the impact of pollution on the performance of solar PV panels. We have proposed a machine learning model based on the Stacking Ensemble classifier to classify the pollution source on the PV panels’ surface. Different sources of pollution are used on each solar panel, and then their power output is recorded. Furthermore, a hybrid model with the random classifier, extra trees, and gradient boost classifier is created. Different weather characteristics were considered during model training. Various evaluation criteria, including precision, accuracy, and the F1 score, were utilized to evaluate the performance of the suggested model. An accuracy of 97.37% was achieved using the proposed model. Additionally, the proposed method was compared to other advanced machine-learning models.

The study’s key finding is that surface pollution can significantly reduce solar panel performance, and appropriate measures are necessary to maintain optimal power output. The proposed machine learning model, which is based on the Stacking Ensemble classifier, can help classify the sources of pollution on PV panels and come up with specific cleaning and maintenance plans to improve the efficiency of the panels. By identifying specific types of pollution affecting the panels’ efficiency, the proposed model can help improve the overall efficiency of the panels and reduce the costs associated with maintaining them by enabling more targeted cleaning and maintenance efforts. The proposed model can also guide the PV panels’ maintenance schedule by identifying when specific sources of pollution need to be cleaned, thus reducing the need for frequent cleaning and maintenance.

Various potential future research directions could be taken to improve the proposed model for classifying pollution sources on photovoltaic (PV) panels. These include incorporating additional and diverse data sources, testing the model in real-world scenarios, and combining it with other techniques, such as image processing, to improve its accuracy and robustness. Explainability methods, like LIME, could be added to the proposed model to help people understand the model’s decision and the factors that affect how well the PV panels work.

In conclusion, the proposed machine learning model can make solar PV panels much more efficient by classifying pollution sources and coming up with targeted cleaning and maintenance plans. The results of this study and the experiments conducted have important implications for how solar panels are maintained and can help make sure they work at their best power output.

## Data Availability

The dataset used and analysed during the current study are available from the corresponding author on reasonable request.
